# Supplementary material for: Sensory cues, behavior and fur-based drying in the rat wetness response
Source: Sci Rep. 2024 Oct 19;14:24550. doi: 10.1038/s41598-024-74900-9 (PMC11490484; doi:10.1038/s41598-024-74900-9)
Supplement: Supplementary file 1 — Supplementary Material 1 [file 41598_2024_74900_MOESM1_ESM.docx]

**Supplementary Material**

**Supplementary Figure 1**


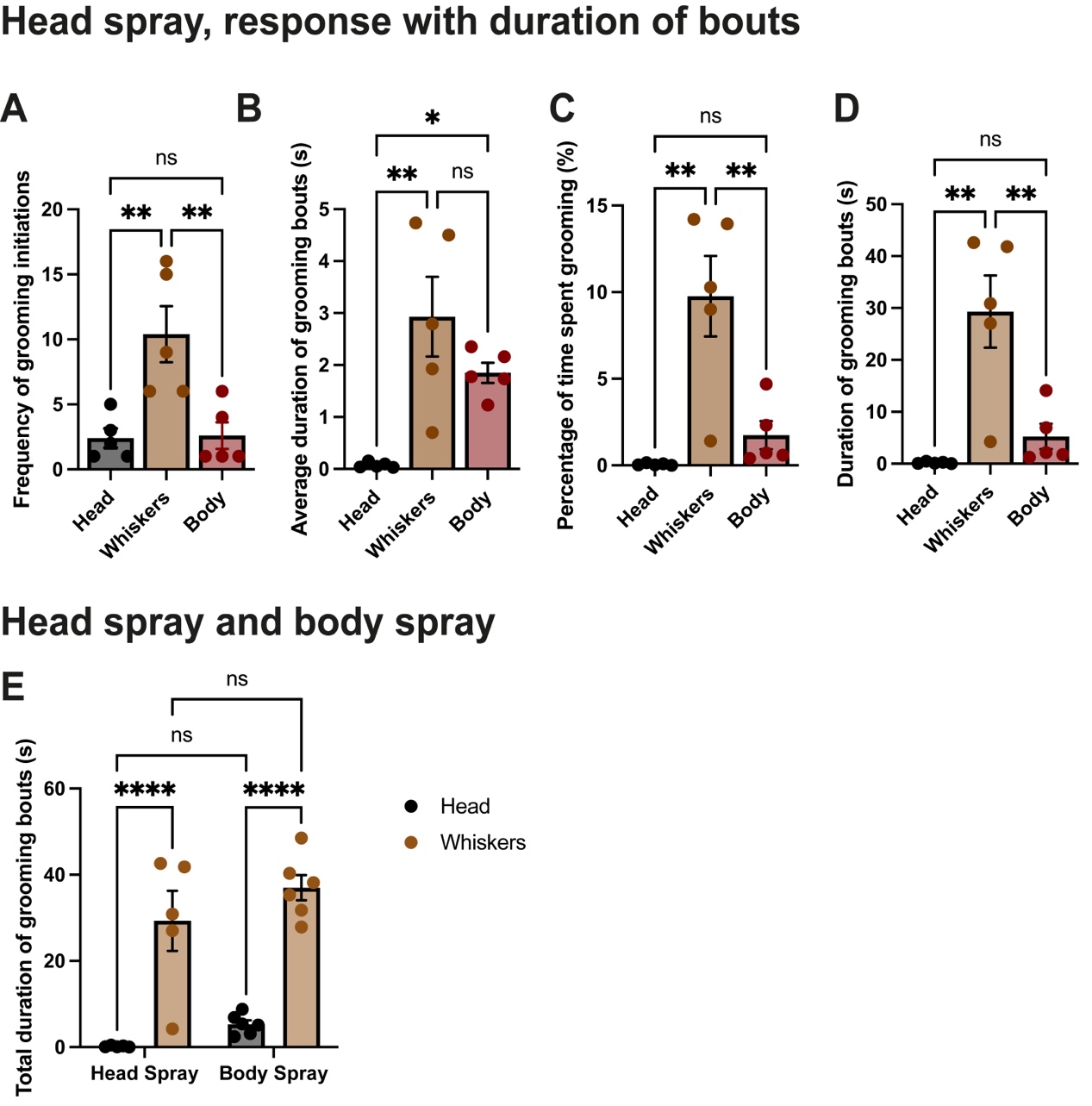


**Figure S1 Duration of whisker, head and body grooming bouts when rats were sprayed on the head. (A)** Frequency of head and whisker grooming initiations when rats were sprayed on the head. (B) Average duration of grooming bouts (seconds). **(C)** Percentage of time spent grooming out of 5-minute trial. **(D)** Total duration of grooming bout, out of 5-minute trial (n= 5 animals). **(E)** Duration of grooming bouts directed towards the head and whiskers following head and body spray (n= 5 animals head spray; n= 6 animals body spray). Bars indicate the mean, error bars indicate ± SEM. Data underlying this figure can be found at

<https://figshare.com/s/db1e981b35d07478a38e>

**Supplementary Figure 2**


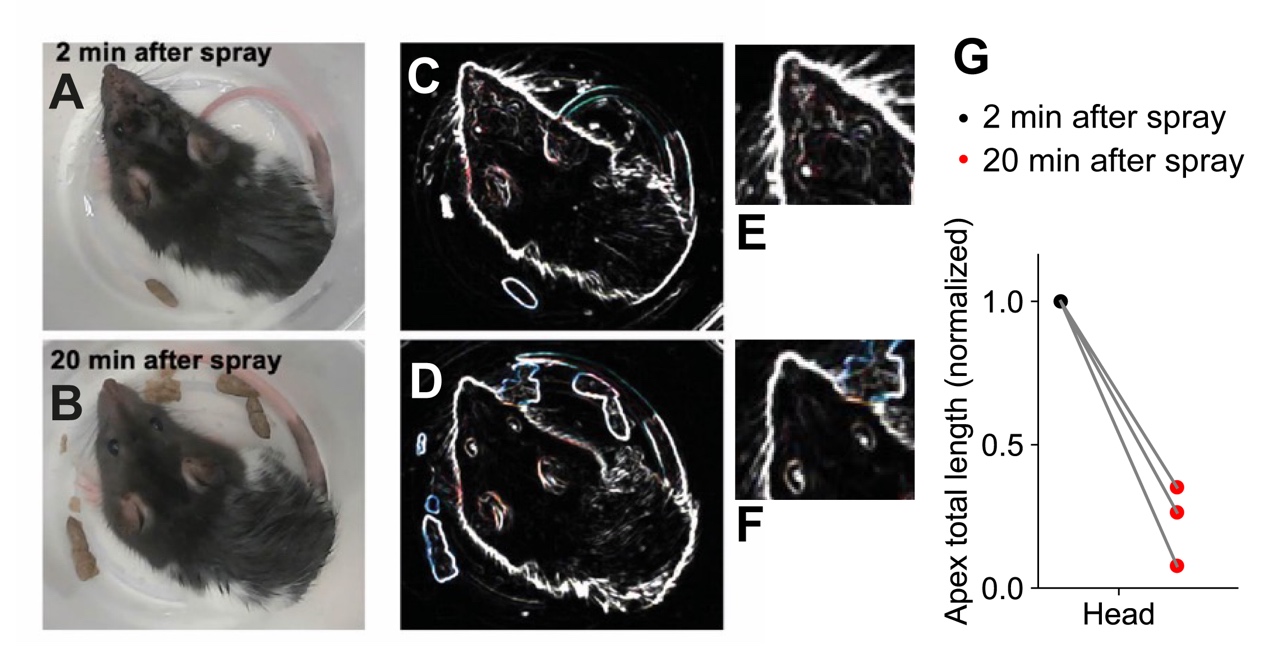


**Figure S2 Quantification of the appearance and disappearance of fur apexes in the drying process.** Photographs of a rat showing (**A**) 2 min and (**B**) 20 min after water spraying on the head and body. Variance filtered **(C)** and **(D)** exemplifying the results of edge detection and apex locations on the head. Images with head magnified (**E-F**). **(G)** We quantified apexes (elongated tapering hair clumps) as the normalized total length of edges (visible as white lines after edge detection) at the 2 min and 20 min after spray (n = 3 animals); the disappearance of apexes as a result of drying is well visible. Data underlying this figure can be found at <https://figshare.com/s/9985a434fd669361cabe>

**Supplementary Figure 3**

**
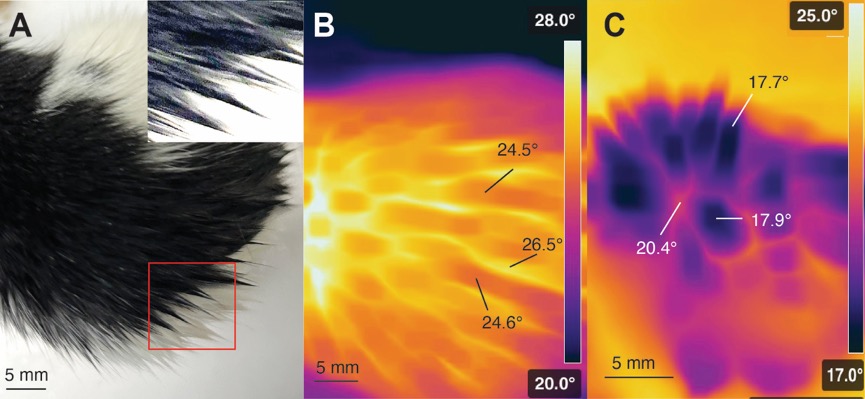
**

**Figure S3 Photography and thermal imaging of wet fur apexes *in vivo* and *in vitro*. (A)** Photograph of the back fur of a wet rat after water spraying as described before. Upper right inset shows fur apexes. **(B)** Thermal imaging of the wet back of the same animal. A regular pattern of colder fur apexes is seen; apexes are 2-3° Celsius colder than in between fur areas. **(C)** Thermal imaging of excised back fur. Apexes were generated by rubbing water into back fur against the laying direction of the fur. As *in vivo* (B) a pattern of colder fur apexes is seen; apexes are 2-3° Celsius colder than in between apex fur areas.
